# The Effect of Polyamide 11 on the Thermal Stability and Light Transmittance of Silicone-Based Thermoplastic Vulcanizates

**DOI:** 10.3390/polym16030324

**Published:** 2024-01-24

**Authors:** Muhammet Iz, Jinhyok Lee, Myungchan Choi, Yumi Yun, Jongwoo Bae

**Affiliations:** Korea Institute of Footwear & Leather Technology, Busan 47154, Republic of Korea; muhammet.iz.90@gmail.com (M.I.); jhlee@kiflt.re.kr (J.L.); mcchoi@kiflt.re.kr (M.C.); ymyun@kiflt.re.kr (Y.Y.)

**Keywords:** polyamide-11, polyurethane, dispersion of silicone rubber, light transmittance, thermal stability

## Abstract

The effect of polyamide 11 (PA11) on the thermal stability and light transmittance properties of silicone-based thermoplastic vulcanizates (TPVs) has been investigated. The blends were prepared through a dynamic vulcanization process by adding 15, 30, and 45 wt% PA11 to the silicon-based TPVs, respectively. The effect of PA11 on the dispersion of silicone rubber in the TPVs after dynamic vulcanization was characterized by a scanning electron microscope (SEM), the thermal stability of the compounds was evaluated through the changes in mechanical performance in the thermo-oxidative aging process, and the light transmittance of TPVs was measured by a haze meter. The results showed that adding PA11 to silicone-based TPVs caused a decrease in the size of the silicone rubber particles after dynamic vulcanization, resulting in improved dispersion. Due to this, by increasing the compatibility between the segments through silicone’s effective dispersion, the amount of light absorption was reduced, and the amount of light transmittance was increased. Finally, according to the results of the thermal aging test, it was found that TPVs with 30 and 45 wt% PA11, respectively, showed outstanding thermal resistance after aging at 160 °C and 168 h and did not melt down.

## 1. Introduction

In the field of polymer processing, the development of new thermoplastic vulcanizates (TPVs) has been particularly active since thermoplastic/rubber blends with controlled structure and morphology can be obtained through dynamic vulcanization [1,2,3,4]. A wide range of TPV material qualities can be tailored depending on the composition and behavior of the dispersed phase. Additionally, TPVs have a number of benefits over conventional crosslinked elastomers, including the ability to be recycled like thermoplastics and achieve functional capabilities comparable to thermoset elastomers utilizing typical polymer melt processing techniques. Moreover, compared to the equivalent rubber or plastic, the resultant composite has more elastomer-like qualities, such as a lower compression set, decreased stiffness, and increased resilience to heat, chemicals, and fatigue [5,6]. However, due to the low heat resistance and poor oil resistance qualities of the thermoplastic matrix, most TPVs have limited usage in automotive applications. Dow Corning created a new series of high-performance TPVs called ‘super TPV’ that are based on cross-linked silicone rubber particles distributed in a variety of engineering thermoplastic matrices to meet these two objectives [7,8]. In the present investigation, a thermoplastic polyurethane (TPU) and silicone rubber (PDMS) TPVs system with a different blend ratio has been used as the base matrix.

TPU is a linear segmented block copolymer containing soft and hard segments. In most TPUs, aliphatic polyether polyols or aliphatic polyester polyols as the soft segments are coupled with hard segments formed from aromatic di-isocyanates and short diols by urethane linkages. Also, it is known that TPUs’ hard segments can form hydrogen bonds with one another, which provides good physical characteristics [9,10,11]. However, it is known that there is still a need to prepare PU compounds that can retain physical properties after exposure to heat (120–150 °C) for extended periods of time.

Due to its well-known distinctive characteristics such as low glass transition temperature and resistance to ultraviolet radiation, PDMS is widely used in various kinds of industrial areas [12]. Its primary chain is made up of extremely flexible O-Si-O bonds with methyl groups bonded to silicon atoms, and it has remarkable thermal stability [13]. As a result of this, PDMS-based TPVs were conceived and developed to improve the performance of general-purpose TPVs under extreme conditions, particularly in automotive under-hood and industrial parts subjected to high temperatures (135–170 °C) [14]. However, silicone rubber exhibits rather poor mechanical properties.

By using TPU and PDMS together, TPVs that contain both the high physical properties of TPU and the high-temperature resistance and flexibility of silicone can be produced. However, due to the difference in the molecular structure between PU and silicone rubber, the blend is immiscible. Due to thermodynamically induced phase separation, the miscibility of thermoplastic polyurethane and silicone rubber can cause a rapid deterioration in the blend properties. Compatibility is, therefore, necessary to achieve good interfacial adhesion and to lessen interfacial tension. Compatibilizers, used as interfacial agents, can be added to immiscible polymer blends to prevent coarse and unstable morphologies caused by high interfacial tension between the phases [15,16,17,18]. By using compatibilizers that reduce the interfacial tension between the two phases, increase the surface area of the dispersed phase, promote adhesion between the phase components, and stabilize the dispersed phase morphology, the physical and mechanical properties of the blends can be greatly improved [19]. The physical properties of TPVs are known to be strongly dependent on the blend composition, the cross-link density of the rubber phase, and the rubber dispersion and particle size [20,21,22,23,24,25,26,27]. Lei et al. [15] prepared PDMS/PU TPVs by using a segmented PU modified by hydroxyalkyl functional PDMS as a compatibilizer, an organohydrido silicone compound as a curing agent, and platinum as a catalyst. The size of the rubber particles dispersed in the PU matrix was less than 8 µm and the elongation at break (EB) of the TPVs reached 942.6%. Papke and Karger-Kocsis [27] demonstrated that a compatibilizer (glycidyl methacrylate-grafted rubber) can increase compatibility and size in poly-ethylene terephthalate and different rubber TPVs. Hu et al. [28] discovered that adding dimethyl silicone oil to the PU/Si-rubber hybrid system reduced the diameter of PU agglomerates and made the distribution of PU agglomerates more uniform. Maity and Khatua [29] applied vinyl triacetoxy silane-grafted silicone rubber to improve the compatibility and physical characteristics of PU/silicone rubber blends, and they discovered that physical properties including hardness, modulus, and tensile strength improved when compared to the reference compound blend. However, with the exception of a few patents describing the production of PU/silicone rubber TPV [30] no study has been published on the structure and properties of this type of TPV. Even in those patents, the system’s compatibility is not explicitly stated.

PA11 is a bio-based polymer, synthesized from castor oil which has good mechanical properties and is capable of withstanding high temperatures. Since intermolecular hydrogen bonds are built between the amide groups of polyamide and the urethane groups of TPU, polyamides are expected to be more compatible with TPU and may even change the final properties of thermoplastic vulcanizates, such as their physical properties and thermal resistance [31]. Zhang et al. [32] mixed TPU with PA6, and they discovered that the impact strength is highest when TPU makes up 20% of the blend weight (wt%). Kim et al. [33] used PA11 to modify the ether- and ester-based TPU’s properties. They discovered that PA11 increased the tensile strength of TPVs without a significant decrease in elongation at break, the crystallization temperature of TPVs increased, and the structure became more homogenous in the continuous phase due to the good dispersion of PA11. In this study, PDMS based-TPU thermoplastic vulcanizates were prepared by adding PA11 to observe compatibility between TPU and PDMS. The relationship between the different materials and the mechanical properties of the blend TPVs was discussed. The effect of PA11 on the thermal resistance and light transmittance of compounds was investigated.

## 2. Materials and Methods

### 2.1. Materials

Ether based TPU 6175AP (specific gravity: 1.08 g/cc, hardness: 79 Shore A, T_m_: 150 °C) was provided by Dongsung Chemical Co., Ltd., Ulsan, Republic of Korea. The bio-based polyamide 11 (Rilsan BECNO TL, T_m_: 189 °C) was purchased from Arkema, France. Epoxy- terminated PDMS gum GC DV SR-E30 (epoxy content of 0.36 wt%, molecular weight: 610,000 g/mol) was provided by Grace Continental Co., Bucheon, Republic of Korea. The chain extender (ADR 4468) and UV stabilizer (Tinuvin 770) were provided by BASF, Seoul, Republic of Korea. The antioxidant (Naugard 445) was purchased from SI Group Inc., New York, NY, USA. 2,5-Dimethyl-2,5-di(tert-butylperoxy) hexane (Triganox 101-50D-PD) was purchased from Nouryon, Amsterdam, Netherlands.

### 2.2. Sample Preparation

To make it easier to feed silicone rubber into the extruder, 50/50 wt% TPU/PDMS masterbatch was prepared by using an internal mixer and then pelletized by using F/R cutting. The mixing speed of the mixer was 30 rpm, the initial temperature was 140 °C, the dump temperature was 160 °C, and the mixing time was under 7 min. Before the dynamic vulcanization process, TPU, PA11, and TPU/PDMS M/B pellets were dried in an oven (TFD2-52-1E1, Despatch, Minneapolis, MN, USA) at 80 °C for 4 h. The dry blending method was used to prepare the batches Table 1, which were then melt-blended in a co-rotational twin-screw extruder (KZW25TW-40/60hg-NH-440, Technovel, Japan) (screw of 25 mm diameter and L/D 60) at 70 rpm. The temperature profile of the twin-screw extruder was divided into ten heating zones and temperature profiles from the feed hopper to the die were 140/160/170/170/180/185/190/195/200/200 °C.

After the dynamic vulcanization process, the pelletized TPVs dried in an oven at 80 °C for 4 h, then samples were made using a plastic injection molding machine (Pro-15WD, Dongshin Co., Ltd., Changwon-si, Republic of Korea) for physical property analysis and further inquiry. The injection machine’s temperature was divided into four heating zones, with temperatures from the feed to the die in the range of 170/180/190/190 °C, and the cooling time was 30 s.

A 0.75 mm thick TPVs sheet was prepared from dried TPVs pellets using a micro-twin-screw extruder (Process 11, Thermo Fisher Scientific Inc., Waltham, MA, USA) to measure light transmittance. To match the color of the material (light gray) to be produced, 10 wt% graphene M/B was added to the pellets prior to the extrusion process. The temperature range of extrusion was set to be the same as in the plastic injection process.

### 2.3. Characterization Methods

The morphology of the TPVs and dispersion of silicone aggregates was investigated by field-emission scanning electron microscopy (FE-SEM; JSM-6701F, JEOL Ltd., Tokyo, Japan) at an accelerating voltage of 15 kV under an N2 atmosphere. Before the observation, the specimens were cryogenically broken after immersion in liquid nitrogen and then coated with gold.

The thermal transition of the TPVs was examined by differential scanning calorimetry (DSC-250, TA Instruments Inc., New Castle, DE, USA) under a nitrogen flow (20 mL min^−1^). Samples were crimple sealed in aluminum pans and heated to 220 °C at a heating rate of 20 °C min^−1^ (first heating scan), equilibrated at 220 °C for 5 min, cooled at 10 °C min^−1^ to −50 °C, equilibrated at −50 °C for 5 min, and then heated again to 200 °C at 10 °C min^−1^ (second heating scan). The glass transition (*T*_g_) and the melting (*T*_m_) temperatures were determined as the midpoint of the change in the slop of the baseline and the maximum melting peak, respectively. These values were determined on the second heating scan since the first scan served to erase the previous thermal history. Percent crystallinity is determined by equation:

$$X_{\mathrm{PA}}\;(\%)=\left(\frac{∆H_{m}}{∆H_{m0}\times W}\;\right)\times 100$$

where **∆***H*_m0_ = 206 J/g for 100% crystalline PA11 [34], W is the PA11 weight percentage in the blend, and **∆***H*_m_ corresponds to the melting enthalpy.

Thermogravimetric analysis (TGA) was conducted by (Universal V4.5A, TA Instruments Inc., New Castle, DE, USA) under the nitrogen atmosphere from room temperature to 800 °C at a heating rate of 10 °C min^−1^.

The interaction between TPU, PDMS, and PA11 was assessed by using ATR-FTIR (Jasco Inc., Easton, MD, USA). The spectra were obtained using an ATR-diamond tool in the range of 650–4000 cm^−1^.

The mechanical properties of vulcanizates were measured using a universal testing machine (UTM, DUT-500 CM, Dae Kyung Engineering Co., Ltd., Bucheon-si, Gyeonggi-do, Republic of Korea), according to test method A in ASTM D412 [35], at room temperature. The dimensions of the test specimens were 100 mm × 25 mm × 2 mm in size. The crosshead speed was 500 mm/min, and at least 3 samples were used to measure the mechanical properties.

The thermal oxidative aging treatment was performed at 160 °C in a convection oven (OF3-15W, Jeio Tech Co., Ltd., Daejeon, Republic of Korea) for 168 h. Then, the mechanical properties of composites were measured under the same conditions as previously described.

The light transmittance of the TPVs samples was measured according to ASTM D1003 standard [36] using a haze meter (Illuminant D65, wavelength: 400~700 nm, vTH-100, CHNSpec Co., Ltd., Hangzhou, China).

## 3. Results

### 3.1. Morphology of TPVs

**Figure 1:**
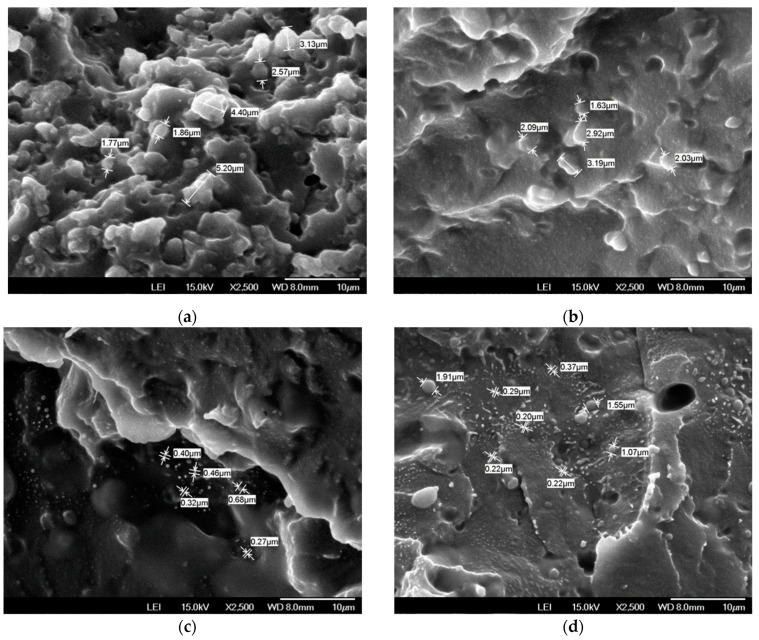
SEM images of fractured surface of blend TPVs: (**a**) PA-0; (**b**) PA-15; (**c**) PA-30; (**d**) PA-45.

Figure 1 shows representative SEM images of silicone-based TPVs. TPVs comprise two or more polymer phases, a continuous phase (TPU) that is hard at room temperature but becomes fluid at high temperatures, while another, dispersed phase (PDMS) is soft and elastic at room temperature [14]. The dispersed phase’s crosslinking degree (CD), its domain size, the rubber particles network’s structure, the thickness of the plastic ligaments, the interface between the rubber and the plastics, and the plastic phase’s crystallinity are all considered to be part of the microstructure of TPVs, and they can all have significant effects on the materials’ properties. In general, a better dispersion in the dispersed phase leads to better physical properties [37,38]. In the case of PA-0 (Figure 1a), it can be clearly seen that the size of the silica particles that come from silicone is bigger than those other pictures. Although the size of the aggregated silica particle decreased when 15 wt% PA11 (Figure 1b) was added to the blend, no discernible difference was seen. However, as the amount of PA11 in the blends increased to 30 wt% and 45 wt% (Figure 1c,d), respectively, silica particle sizes were observed to decrease to under 1.0 μm. SEM analysis results show that adding PA11 to the TPU/PDMS blend increases the dispersion of PDMS in the TPU matrix.

### 3.2. Differential Scanning Calorimetry (DSC) and Thermogravimetric Analysis (TGA)

**Figure 2:**
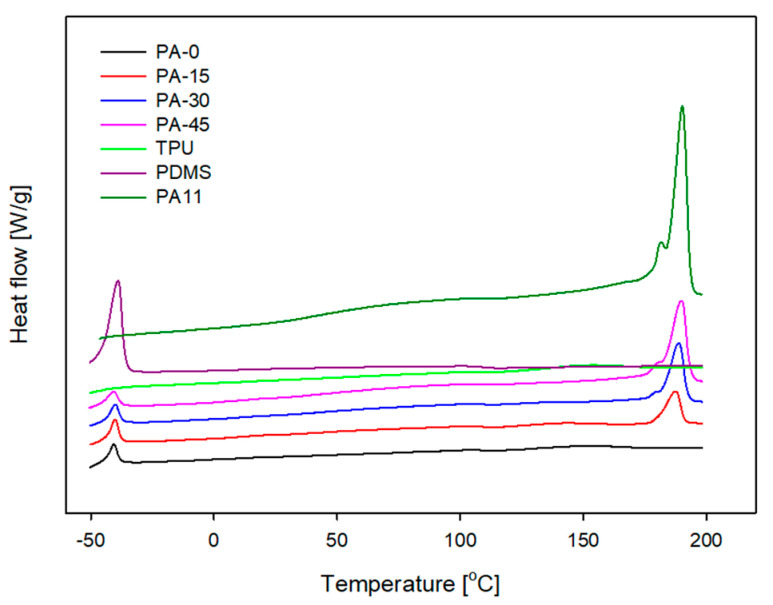
DSC melting thermogram of the neat materials and blend TPVs.

DSC curves and the melting behavior for blend TPVs and PA11 are shown in Figure 2 and Table 2, respectively. The melting temperature and melting enthalpy of the TPVs were determined from the second heating scan after a preliminary heating scan to clear up past thermal history.

The DSC curve of PDMS showed that the melting point (*T*_m_) of the PDMS was at −40 °C, which was found to be similar for all blended TPVs. The *T*_m_ of PA11 is 189.3 °C, and a shoulder peak appears at the maximum melting point. The appearance of the shoulder could be attributed to the development of two separate crystal morphologies in the PA11 polymer or to the behavior of recrystallization after the first heating cycle [39].

Endothermic behaviors observed during differential scanning calorimetry imply the disordering of crystallites. Endothermic peaks at low temperatures are associated with crystallites with a relatively short-range order, which can be created by annealing, for example; meanwhile, the long-range ordered disordering of hard segment crystallites is responsible for the endothermic peaks observed at high temperatures. The small peak at approximately 148 °C, which occurs on the DSC curve of PA-0, is related to the disordering of crystallites with relatively short-range orders. This peak indicates that the material has a low degree of crystallization, making it comparatively soft. The hardness relationship between TPVs will be shown later in the mechanical properties section. The temperature at which this peak occurs is shown to increase to 158 °C with the addition of PA11 and to disappear with increasing PA11 concentration [40]. The appearance of a single endotherm in the blend TPVs as PA11 content increases can be explained by hydrogen bonding interactions between the amide group of PA11 and the urethane/urea group TPU. Due to their chemical structures and the availability of functional groups, PA11 and TPU can develop massive hydrogen bonds. These two polymers, due to extensive hydrogen bonding, develop strong interactions during melting and spinning, thus affecting the morphological structure of blend TPVs which also increases the associated degree of crystallinity (X_PA_) of PA11 [41].

**Figure 3:**
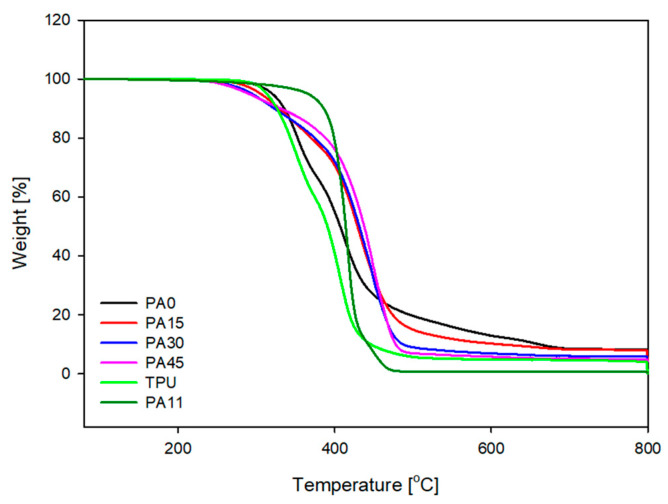
TGA curves for the neat materials and blend TPVs.

In addition to the DSC analysis, TGA analysis was carried out to confirm the effect of PA11 on the thermal characteristics of silicone-based TPU thermoplastic vulcanizates. The TGA analysis curve of the neat materials and blend TPVs and the degradation temperatures are shown in Figure 3 and Table 3, respectively. The T_d_, T_20_, T_50_, and T_75_ temperatures represent the initial temperature at which the degradation of the materials begins and the temperatures at which they complete 20%, 50%, and 75% degradation, respectively. Table 3 shows that the initial degradation temperature of the PA-0, which has no presence of PA11, is surprisingly higher than that of the other TPVs that do contain PA11. However, as the test temperature begins to rise, it is observed that the degradation rate of the PA-0, which does not contain PA11, is faster than the composites containing PA11. Moreover, it was observed that, as the amount of PA11 increased, the temperature required for the degradation of 20%, 50%, and 75% of the TPVs also increased.

### 3.3. Attenuated Total Reflection Fourier-Transform Infrared Spectroscopy (ATR-FTIR)

**Figure 4:**
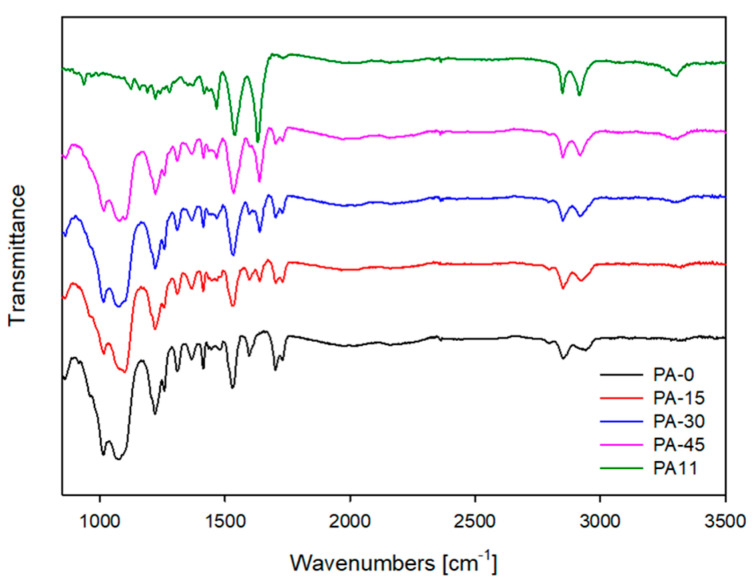
ATR-FTIR spectra of neat PA11 and blend TPVs.

In order to investigate the possible interactions between materials, ATR–FTIR analyses were carried out and are shown in Figure 4. The PA11 components (N-H (3306 cm^−1^), C-H (2917 and 2847 cm^−1^), amide I (1631 cm^−1^), and amide II (1540 cm^−1^)), as well as the TPU(PA-0) components (C=O (1699 cm^−1^), were observed in the blend TPVs.

The carbonyl group (C=O, 1699 cm^−1^) that was observed in the PA-0, slightly shifted to 1700 cm^−1^, 1701 cm^−1^, and 1703 cm^−1^ for PA-15, PA-30, and PA-45, respectively. The crystalline regions of urethane and urea groups can be connected to the carbonyl region of TPU [34]. In PA11-added TPV blends, free urea (keto group C=O) in TPU transforms to H-bonded urethane. This peak’s shift can be attributed to hydrogen bonding interactions between the urea group of TPU and the amide group of PA11 [42,43].

### 3.4. Light Transmittance

**Figure 5:**
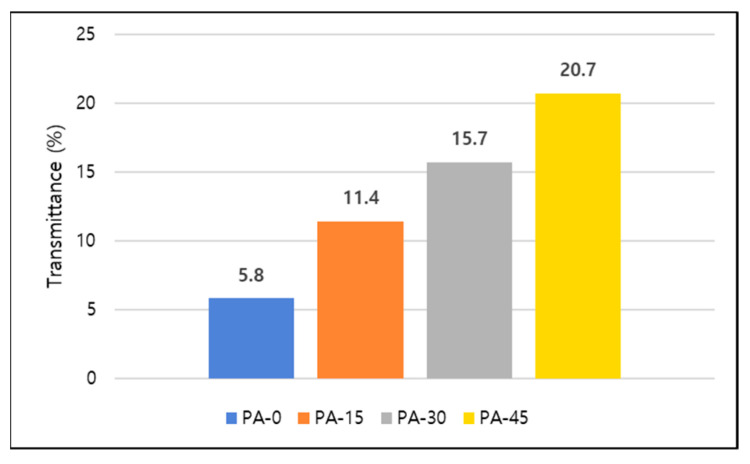
Light transmittance of blend TPVs.

The light transmittance of TPVs was investigated using a haze meter. Transmittance is the amount of light that can flow through a material without being reflected or absorbed and the light transmittance of the TPVs was measured as 5.8%, 11.4%, 15.7%, and 20.7% for PA-0, PA-15, PA-30, and PA-45, respectively, as seen in Figure 5. When it comes to the light transmittance of TPVs, the compatibility of the materials is quite important. Furthermore, phase separation and the transparency of polyurethane materials are known to be influenced by the diisocyanate’s chemical structure, such as the kind and molecular weight of the soft segment, the degree of crystallinity, and intermolecular interactions (hydrogen bonding) [44,45]. In conclusion, light transmittance will be high if the compatibility between the continuous phase (TPU) and dispersed phase (PDMS) of TPVs is high [46].

**Figure 6:**
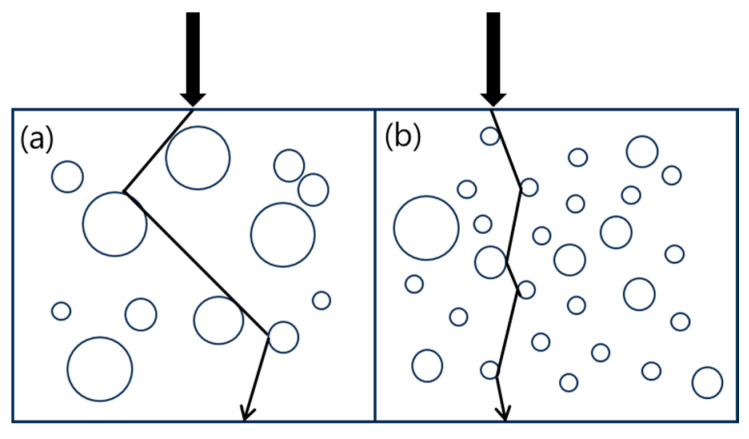
Representative scheme of transmission of light through the TPVs: (**a**) blend TPVs without PA11; (**b**) blend TPVs with PA11.

In other words, a longer path means more time for light to be absorbed. As seen in Figure 6, as the size of the dispersed PDMS particle increases, the path of the light before leaving the material becomes longer, which causes a decrease in light transmittance. According to the SEM analysis results, it was observed that as the amount of PA11 in TPVs increased, the particle size of PDMS decreased. This caused the light transmittance of the TPVs to increase.

### 3.5. Mechanical and Thermo-Oxidative Aging Properties

**Figure 7:**
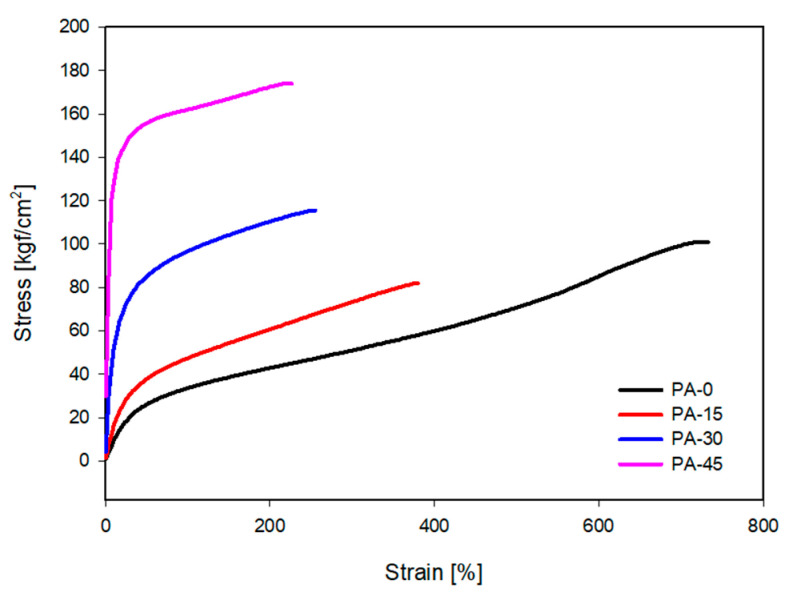
Mechanical properties of blend TPVs.

Figure 7 and Table 4 represent the mechanical properties of TPU/PDMS TPVs mixed with various amounts of PA11. It was observed that, with the increase in PA11 content, the shore A hardness and modulus at 100% of TPVs increase. The mechanical properties of TPVs depend on the size and size distribution of the rubber domains as well as the compatibility between the plastic and rubber phases [47,48,49,50]. As previously indicated, PA11 enhanced the dispersion of PDMS in the TPU matrix and the TPU/PDMS blend’s compatibility, hence improving the mechanical properties of the TPVs.

**Figure 8:**
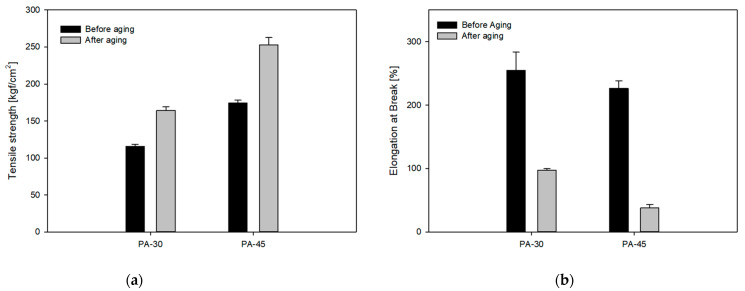
Thermo-oxidative aging properties of blend TPVs: (**a**) tensile strength; (**b**) elongation at break.

The aging performance of blend TPVs is evaluated by the change ratio of the hardness, tensile strength (TS), and change ratio of EB, which are shown in Figure 8a,b and Table 5, respectively. The heat resistance of the PA-0 and PA-15 blend TPVs could not be measured since they melted after two hours of being placed in a 160 °C oven. However, the PA-30 and PA-45 blend TPVs maintained their shape for 168 h, and only a change in color was observed. Moreover, these two blend TPVs maintained the hardness and showed minor changes. As PA11 has a high melting temperature, it forms hydrogen bonds with the hard segment of TPU, and these bonds increase as the amount of PA11 increases. As a result, the PA-30 and PA-45 blend TPVs can endure temperatures of 160 °C.

## 4. Conclusions

Different amounts of PA11 from 0 wt% to 45 wt% were added to investigate the effect of PA11 on the thermal oxidation resistance and light transmittance of silicon-based TPVs, and the compatibility between silicone rubber and thermoplastic polyurethane. It was found that, when the amount of PA11 in the blend increases, the particle size of PDMS decreases, thus the compatibility between the continuous phase (TPU) and dispersed phase (PDMS) of TPVs increases. DSC and ATR-FTIR analyses showed that hydrogen bonding was formed between the urea groups of TPU and the amide groups of PA11, which had an effect by increasing the dispersion of PDMS. It was also discovered that the mechanical properties of TPVs such as hardness, tensile strength, and modulus at 100% are improved as a result of better compatibility between silicone rubber and thermoplastic polyurethane, while elongation at break is significantly decreased. Furthermore, because PA11 has a high melting temperature, and forms hydrogen bonds with the hard segment of TPU, the PA-30, and PA-45 blend TPVs can endure temperatures of 160 °C. Finally, as PDMS size in the TPVs reduces, the path of light before leaving the material shortens, resulting in enhanced light transmittance.

## Figures and Tables

**Table 1:** Recipe of blend TPVs (unit: wt%).

Sample	PA-0	PA-15	PA-30	PA-45
TPU	60	45	30	15
PA11	-	15	30	45
TPU/PDMS M/B ^1^	40	40	40	40
Chain extender	0.5			
UV stabilizer	0.3			
Anti-oxidant	0.5			
Triganox 101-50D ^2^	0.5			

^1^ 50/50 wt% TPU-PDMS masterbatch; ^2^ 2,5-Dimethyl-2,5-di(tert-butylperoxy) hexane.

**Table 2:** Thermal characteristics of neat materials and blend TPVs.

Sample	*T*_m (TPU)_ (°C)	*T*_m (PA11)_ (°C)	∆*H*_m (PA11)_ (J/g)	X_PA_ (%)
PA11	-	190.1	54.0	26.2
PA-0	148.5	-	-	-
PA-15	154.3	187.6	4.6	14.9
PA-30	-	188.7	9.7	15.7
PA-45	-	189.9	17.6	19.0

**Table 3:** Thermal properties of neat materials and blend TPVs.

Sample	*T*_d_ (°C)	*T*_20_ (°C)	*T*_50_ (°C)	*T*_75_ (°C)
PA-0	320	352	406	458
PA-15	305	370	429	462
PA-30	294	374	431	464
PA-45	290	388	439	465

**Table 4:** Mechanical properties of blend TPVs.

Mechanical Properties	PA-0	PA-15	PA-30	PA-45
Hardness (Shore A)	73.0	78.0	89.09	96.0
Tensile strength (kgf/cm^2^)	108.4	81.9	115.6	174.1
Elongation at break (%)	736.8	379.8	254.9	226.6
Modulus at 100% (kgf/cm^2^)	33.7	47.4	96.9	162.1

**Table 5:** Mechanical properties of blend TPVs after thermo-oxidative aging.

After Aging (168 h 160 °C)	PA-0	PA-15	PA-30	PA-45
Hardness (Shore A)	-	-	91.0	97.0
Tensile strength (kgf/cm^2^)	-	-	164.1	252.9
Elongation at break (%)	-	-	41.9	37.9
Change in hardness (Shore A) (pts)	-	-	2.0	1.0
Change in tensile strength (%)	-	-	41.9	45.3
Change in elongation at break (%)	-	-	−61.9	−83.3

## Data Availability

Data are contained within the article.
